# Suzuki-Miyaura Cross-Coupling in Acylation Reactions, Scope and Recent Developments

**DOI:** 10.3390/molecules18011188

**Published:** 2013-01-17

**Authors:** Marco Blangetti, Heléna Rosso, Cristina Prandi, Annamaria Deagostino, Paolo Venturello

**Affiliations:** 1 Centre for Synthesis and Chemical Biology, School of Chemistry and Chemical Biology, University College Dublin, Belfield, Dublin 4, Ireland; E-Mail: marco.blangetti@ucd.ie; 2 Chemistry Department, Turin University, via P. Giuria 7, 10125 Torino, Italy; E-Mails: helena.rosso@unito.it (H.R.); annamaria.deagostino@unito.it (A.D.); paolo.venturello@unito.it (P.V.)

**Keywords:** Suzuki-Miyaura cross-coupling, acylation, palladium, ketones

## Abstract

Since the first report and due to its handiness and wide scope, the Suzuki-Miyaura (SM) cross coupling reaction has become a routine methodology in many laboratories worldwide. With respect to other common transition metal catalyzed cross couplings, the SM reaction has been so far less exploited as a tool to introduce an acyl function into a specific substrate. In this review, the various approaches found in the literature will be considered, starting from the direct SM acylative coupling to the recent developments of cross coupling between boronates and acyl chlorides or anhydrides. Special attention will be dedicated to the use of masked acyl boronates, alkoxy styryl and alkoxy dienyl boronates as coupling partners. A final section will be then focused on the acyl SM reaction as key synthetic step in the framework of natural products synthesis.

## 1. Introduction

The introduction of acyl functionalities represents a fruitful and widespread tool to construct building blocks for the synthesis of natural products and pharmaceutical compounds, and a number of approaches are well known among the synthetic organic chemistry community [1,2]. Traditionally, one potential approach is the Friedel-Crafts acylation of substituted aromatic rings [3,4,5]. However, this method entails some problems and limitations such as drastic reaction conditions, low regioselectivity, and a large amount of by-products [6]. Besides, the nucleophilic addition of organometallic compounds to carboxylic acid derivatives can be used to prepare ketones. Organotin [7], zinc [8], cadmium [9], Grignard or organolithium reagents have been successfully used as nucleophilic reagents [10], but low yields are often obtained because of tertiary alcohol formation [11]. The strategies using organoboron compound–mediated cross-coupling processes have been utilized most widely by synthetic chemists because of their non-toxicity and thermal, air, and moisture stability, and can be considered as a readily available method for new carbon–carbon bond formation in organic synthesis [12,13,14]. Suzuki-Miyaura cross-coupling has been successfully exploited to introduce an acyl function onto a specific substrate with high regioselectivity. To this purpose three different approaches can be envisaged. The first of them is the palladium-catalyzed cross-coupling reaction of arylboronic acids with carboxylic acid derivatives (see Section 2), and are based on the cleavage of the C–O bond of the carboxylic derivative in the presence of palladium catalyst. This approach is superior to the previous methods in terms of reaction conditions, efficiency, and functional group compatibility. The second one (Section 3) relies on the carbonylative version of the SM reaction. In most cases 1 atm of CO (g) and mild conditions are sufficient to promote the carbonylative coupling. The last approach (Section 4) deals with the use of masked acyl functionalities, namely masked acyl boronic esters (alkoxy dienylboronates, alkoxy styryl boronates), following a traditional cross coupling reaction with a coupling partner the original acyl function can be easily restored. The intent of this short review is to give a general overview of the most recent advances in the literature in this field. 

## 2. Suzuki-Miyaura Coupling of Acyl Chlorides and Anhydrides

### 2.1. Coupling of Arylboronic Acid Derivatives with Acyl Chlorides

**Scheme 1:**
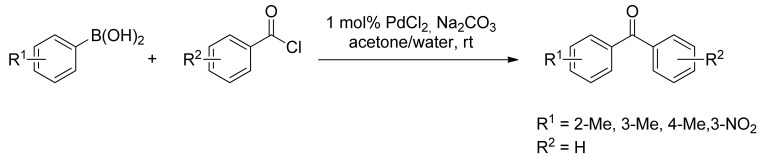
Bumagin’s first carbon monoxide free carbonylation of arylboronic acids.

Many synthetic methods have been developed in the past for the synthesis of diaryl ketones, which are important building blocks in a large number of natural products and active pharmaceutical compounds [15,16,17]. Among them, the conversion of carboxylic acid derivatives into aryl ketones has proven to be a widely employed transformation in the natural product synthesis as well in drug discovery design processes. The most common synthetic approach to aromatic ketones is undoubtedly the Friedel-Crafts acylation of an arene [18]. However, due to its limited regioselectivity and to the incompatibility of certain functional groups, this approach often leads to hard to resolve isomeric mixtures. Moreover, the synthesis of ketones from organometallic reagents and acyl chlorides or esters often proceeds in low yield because of the addition of the organometallic reagent to the ketone to form tertiary alcohols [11,19]. Thus, the necessity of alternative approaches to the synthesis of diaryl ketones embraced the exponential growth of palladium catalyzed methods with the seminal report by Bumagin and coworkers in 1997 [20], where acyl chlorides were reacted for the first time with arylboronic acids in the presence of ligand-free palladium to form the corresponding aromatic ketones in high yields (Scheme 1). The reaction proceeded quantitatively with catalytic PdCl_2_ between arylboronic acids or Ph_4_BNa and acyl chlorides in the presence of Na_2_CO_3_ in aqueous acetone, while easily hydrolyzed acyl chlorides reacted only smoothly with Pd(OAc)_2_ in anhydrous acetone.

**Scheme 2:**
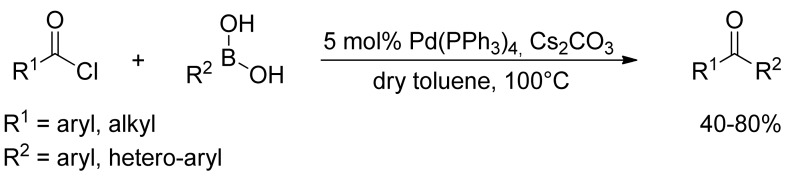
Haddach’s carbonylation of arylboronic acids under anhydrous conditions.

The classic Suzuki coupling reaction, which is generally carried out as a palladium-catalyzed reaction in organic solvent and water, has proven to be poorly suitable for the coupling of acyl chlorides with boronic acids. However, Haddach and coworkers [21] reported in 1999 as an alternative method the first palladium-catalyzed coupling of organoboronic acids with acyl chlorides under anhydrous reaction conditions (Scheme 2). By means of catalytic Pd(PPh_3_)_4_ and cesium carbonate in dry toluene, several symmetric and non-symmetric aromatic ketones have been synthesized in moderate to good yields.

**Scheme 3:**
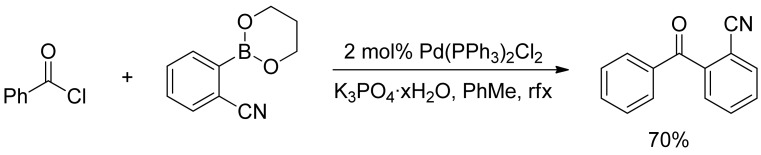
Urawa’s synthesis of *ortho*-cyanobenzophenone derivatives.

In a similar report, Bumagin and Korolev described the phosphine-free palladium-catalyzed acyldeboration of sodium tetraarylborates and arylboronic acids with acyl chlorides in dry acetone in the presence of Na_2_CO_3_ as a base and Pd(OAc)_2_ (1 mol%) as the catalyst precursor [22]. Few years later, Urawa and Ogura found that both Haddach’s [21] and Bumagin’s [22] anhydrous conditions were not suitable in the synthesis of *ortho*-cyanobenzophenone derivatives [23], where only low conversion were achieved. However, they reported in 2003 that the use of K_3_PO_4_ hydrate as a base in the reaction of boronic acids with acyl chlorides in toluene afforded the desired cyanobenzophenone in good yield, and the methodology was successfully applied to the synthesis of symmetric and asymmetric ketones in good to excellent yields (Scheme 3) [23].

**Scheme 4:**
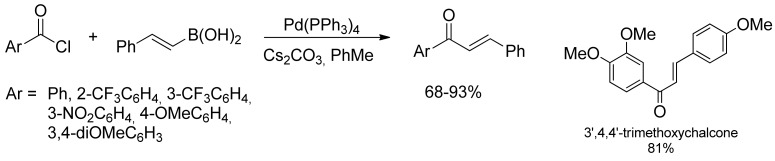
Rolando’s approach to substituted chalcones derivatives by SM-coupling of cinnamyl boronic acids.

At the same time, Rolando and coworkers successfully applied Haddach’s conditions [21] in the coupling reaction between different aryl chlorides and substituted cinnamoyl chlorides leading to a variety of substituted chalcones in near quantitative yield [24]. This approach allowed the access to naturally occurring chalcones starting from suitably methoxylated substrates, as reported in Scheme 4.

**Scheme 5:**
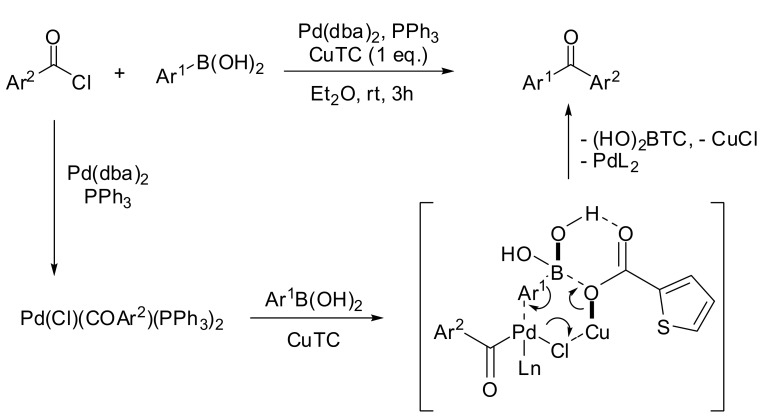
Copper (I)-Pd(II) promoted carbonylation of arylboronic acids.

Several alternative procedures for the SM direct acylation of arylboronic acids have been investigated since the preliminary reports described above. For example, aromatic ketones were synthesized via a palladium catalyzed cross-coupling reaction of boronic acids with acyl chlorides in the presence of catalytic PdCl_2_ and dry Na_2_CO_3_ at room temperature under solvent-free conditions [25]. At the same time, Nishihara and colleagues reported the synthesis of unsymmetrical aromatic ketones by a Pd(dba)_2_ catalyzed reaction in diethyl ether in the presence of copper(I) thiophene-2-carboxylate (CuTC) as an activator [26]. These conditions were successfully applied to a wide range of substrates bearing an electron-donating or an electron-withdrawing substituent. The mechanism proposed by the authors involves a preliminary transmetalation of the acyl palladium(II) chloride by a six-membered transition state, where the soft Cu(I) ion accelerates the polarization of the Pd–Cl bond while the hard carboxylate counterion activates the boronic acid (see Scheme 5).

Microwave-assisted methodologies have also been recently developed providing the ketones in high yields within short reaction times. Simple catalytic systems for cross-coupling reactions of acyl chlorides with arylboronic acids under microwave conditions (260 W, 98 °C, 10 min) were tested by Polàckovà and coworkers in 2006 [27], whereby Pd(PPh_3_)_4_ and Cs_2_CO_3_ were employed as catalytic system for the coupling in wet toluene under irradiation, affording ketones in good to high yields. In 2008 Wolf and coworkers described the SM cross-coupling of aromatic and aliphatic acyl chlorides with arylboronic acids in the presence of 2.5 mol% of (*t*-Bu_2_POH)_2_PdCl_2_ which was completed within 10 min under microwave irradiation (100 W, 80 °C), leading to benzophenone and acetophenone derivatives in good to high yields [28].

**Scheme 6:**
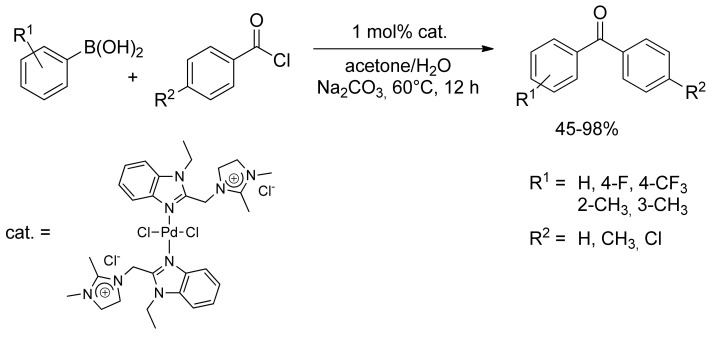
Li’s imidazolium catalyst applied to the synthesis of diaryl ketones.

More recently, new palladium catalysts have been employed in the SM acylation of boronic acid derivatives, as reported by Li and collegues in 2011 [29]. An imidazolium chloride palladium(II) complex has proven to be active toward cross-coupling of arylboronic acid and benzoyl chloride in aqueous acetone solution, giving high yield of the desired aryl ketones (Scheme 6). Noteworthy, the phosphine-free (N–N)Pd(II) complex has been efficiently recycled more than four times almost without any loss of reactivity in the aqueous SM coupling reaction.

### 2.2. Coupling of Arylboronic Derivatives with Anhydrides

**Scheme 7:**
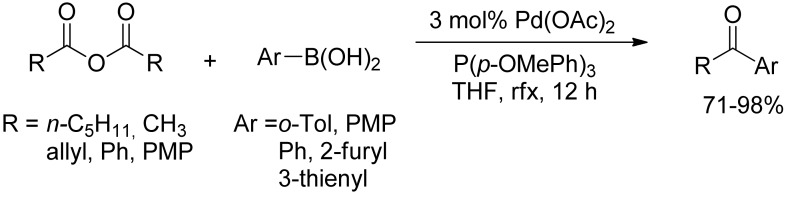
Gooßen’s SM-coupling of arylboronic acids with anhydrides.

Despite the wide amount of diaryl ketones syntheses by SM-coupling reaction of arylboronic acids with acyl halides, the first development of a catalytic system capable of coupling anhydrides with boronic acids has been investigated only at the very beginning of the XXIst century with two independent reports by Yamamoto [30,31,32] and Gooßen [33]. The catalytic process described by the authors consists of the oxidative addition of the anhydride to an acyl palladium complex, exchange of the carboxylate for the aryl group by transmetalation of the boronic acid followed by reductive elimination of the ketone. The reactions were easily performed with many functionalized derivatives (Scheme 7), only required commercially available nontoxic chemicals, and produced a minimum amount of waste, thus making this approach superior to the previous methods in terms of mildness of reaction conditions, selectivity, and efficiency.

**Scheme 8:**
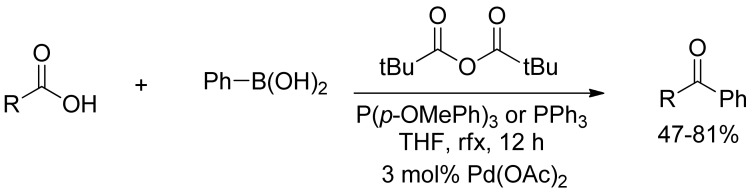
Gooßen’s pivalic anhydride promoted coupling of arylboronic acids with carboxylic acids.

Moreover, Gooßen and coworkers reported an efficient method for the catalyzed SM-cross coupling of carboxylic acids to arylboronic acids in the presence of palladium(II) acetate (Scheme 8) [34]. This nicely described approach combined the activation and cross-coupling step of the carboxylic with boronic acids into a convenient one-pot procedure using pivalic anhydride as an activating agent, without any formation of *tert*-butylated ketones and formation of only pivalic acid and boric acid as side products.

**Scheme 9:**
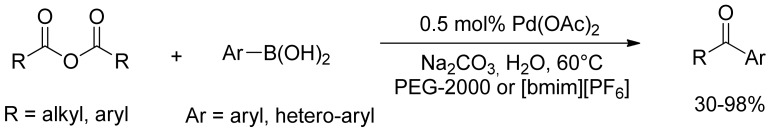
Zhang’s coupling of arylboronic acids with anhydrides in the presence of additives.

However, both Yamamoto’s and Gooßen’s studies showed that the phosphine ligands played a key role in the successful execution of the reactions, and no ketone product was obtained in the absence of phosphine ligands. Thus, the Pd(OAc)_2_ catalyzed coupling reaction of arylboronic acid with carboxylic anhydrides without phosphorous ligands was successfully investigated few years later. In 2006, Zhang and coworkers [35] turned out that this SM-cross coupling could be carried out smoothly in water in the presence of PEG or [bmim][PF_6_] to give high yields of ketones without the use of phosphine ligands with a good recycling of the catalytic system (Scheme 9). Moreover, the authors reported in 2007 that the presence of surfactants has a significant effect on the activity of the cross-coupling reaction in water in the absence of phosphine ligands [36]. Even the effect of acetone as a cosolvent in water was studied in 2008 by Xin [37], showing that a high efficiency without other additives and ligands can be achieved making the method useful and attractive for the synthesis of aryl ketones.

**Figure 1:**
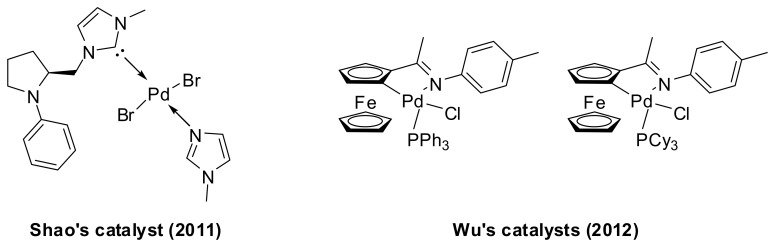
Shao’s (left) and Wu’s (right) catalysts applied in the synthesis of aromatic ketones by Pd-catalyzed coupling of arylboronic acids with anhydrides.

Traditional palladium catalysts for the coupling of arylboronic acids with anhydrides have been recently successfully replaced by new generation catalysts. For example, the use of coordinated monodentate NHC–Pd(II) complex derived from *N*-phenyl substituted proline has been reported by Shao in 2011 in the coupling reaction of arylboronic acids with benzoic anhydride in water [38], and cyclopalladated ferrocenylimines catalysts have been recently explored by Wu and coworkers [39] in the cross-coupling of arylboronic acids with carboxylic anhydrides (Figure 1).

### 2.3. Coupling with Chloroformates and Carbamoyl Chlorides

Since aryl carboxylic esters and aryl carboxamides are useful building blocks both for laboratory synthesis as well as industrial processes [40], several synthetic methods for preparing these compounds have been developed in the past. Among them, metal-catalyzed carbonylation of aryl halides using carbon monoxide as the carbonyl source in the presence of amines or alcohols is the most popular way [41]. To avoid the use of toxic gaseous carbon monoxide, modified processes have been described by using transition metals complexes like nickel, molybdenum and iron as solid sources of monoxide [42,43,44]. Another attractive method for the synthesis of aryl carboxamides is the direct amidation of organometallic reagents with formamide derivatives, even if the organometallic reagents used for such an approach have been limited mainly to magnesium or lithium reagents [45,46,47,48].

**Scheme 10:**
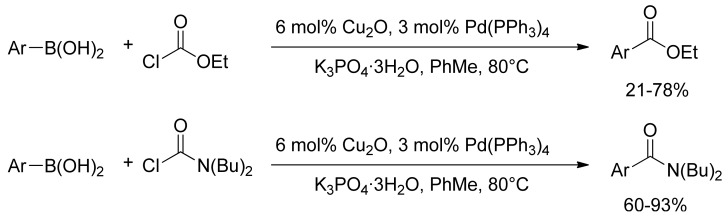
Pd/Cu catalyzed carboxylation and amidation of arylboronic acids derivatives.

The first description of a palladium catalyzed reaction for the synthesis of this class of compounds has been reported by Deng and Duan in 2005 [49]. A convenient preparation of arylcarboxylic esters and arylcarboxamides has been described from arylboronic acids under mild conditions by means of a Suzuki-type cross-coupling reaction of arylboronic acids with ethyl chloroformate or *N*,*N*-dibutylcarbamoyl chloride in the presence of catalytic amounts of Cu_2_O and Pd(PPh_3_)_4_ (Scheme 10).

**Scheme 11:**
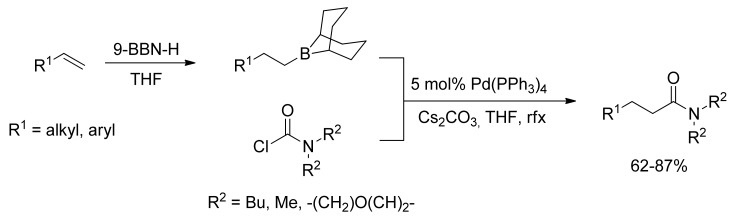
Takemoto’s Pd-catalyzed amidation of olefins via 9-BBN intermediates.

In 2007 Takemoto and coworkers reported a one-pot amidation of olefins through the first palladium-catalyzed coupling reaction of carbamoyl chlorides with alkylboranes [50]. Hydroboration of olefins and subsequent coupling of the resulting alkylboranes with carbamoyl chlorides have proven to be a promising synthetic route for the generation of amides starting from unactivated alkenes (Scheme 11). Moreover, carbamoyl chlorides have been successfully coupled with arylboronic acids in a copper-free Suzuki coupling reaction in the presence of catalytic Pd(PPh_3_)_4_.

**Scheme 12:**
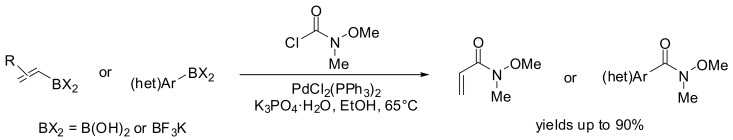
Synthesis of Weinreb amides by SM-coupling of arylboronic acids/ organotrifluoro borates with *N*-methoxy-*N*-methyl-carbamoyl chloride.

More recently, the Sukuzi-Miyaura cross-coupling of commercially available *N*-methoxy-*N*-methyl-carbamoyl chloride with both (hetero)aryl and alkenyl boronic acids or trifluoroborates has been investigated by Herr and coworkers (Scheme 12) [51]. This methodology has proven to be an improvement upon existing methods for the preparation of Weinreb amides, avoiding the use of toxic carbon monoxide in case of the metal catalyzed cross-coupling reactions approach.

**Scheme 13:**
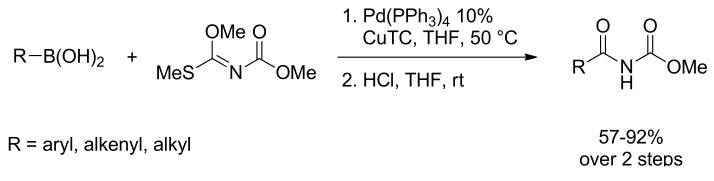
SM cross coupling of Methyl N-[Methoxy(methylthio)methylene]carbamate.

Very recently the preparation of imides via the palladium-catalyzed coupling reaction of aryl-, alkyl-, and alkenylboronic acids with *N*-[methoxy(methylthio)methylene]carbamate in the presence of Cu(I) thiophene-2-carboxylate (CuTC) has been described. The reaction afforded imino ethers that were easily converted to the corresponding imides by acidic hydrolysis in high yield. (Scheme 13) [52].

## 3. Carbonylative SM Reactions

Despite the direct acylation of organoboron derivatives has proved to be an attractive approach to the formation of symmetric and unsymmetrically substituted ketones, the carbonylative Pd-catalyzed coupling reaction has been widely employed for the synthesis of aromatic ketones [12]. Depending on the substrates used, catalytic carbonylation leads to a variety of carbonyl-containing compounds and dicarbonyl compounds. As mentioned above, diaryl ketones are target molecules of importance for organic synthesis since the diarylketo moiety occurs in many natural and synthetic biologically active molecules, as well as in non-steroid anti-inflamatory drugs [53]. Aryl halides with various coupling partners have found success including stannanes [54,55,56], magnesium [57], aluminum [58], and silane derivatives [59], as well as boronic acids [60,61].

However, the application of these methods is limited due to the side reaction which prevent the insertion of carbon monoxide into the ArPdX intermediates and promotes the formation of the Ar-Pd-Ar species to give biaryls. The use of pressured carbon monoxide is a general method for suppressing such a side reaction, which was applied in the reaction of aryltin derivatives with aryltriflates. Relatively high temperature conditions must be used to induce this kind of carbonylative coupling, and the poor reactivity of electron-deficient aryl halides makes this type of reaction less accessible. Moreover, the form of the palladium catalyst, ligands, base, additives, solvent, and temperature have been shown to have an effect on the ketone:biaryl ratio.

α,β-Unsaturated ketones are important key reagents in organic synthesis, as they are widely used as Michael acceptors in conjugated reactions [62,63,64], as activated olefins in Heck-type couplings [65] or in epoxidation reactions [66]. The most common synthetic approach proceeds through an aldol condensation reaction between one equivalent of ketone and one equivalent of an aldehyde derivative followed by a dehydration step. The reaction is extremely efficient especially for the synthesis of chalcone derivatives. Other alternative strategies have been developed to access aryl α,β-unsaturated ketones and among them, the most efficient ones has proven to be the selective oxidation of an allylic alcohol [67] and Friedel-Crafts acylation reaction of arenes using α,β-unsaturated acyl halides [68,69].

**Scheme 14:**
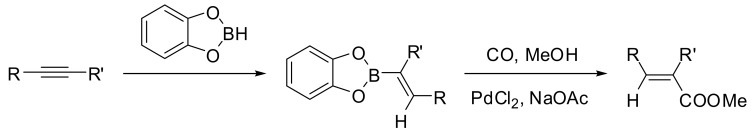
Suzuki-Miyaura carbomethoxylation of alkenylborane derivatives.

While the synthesis of diaryl ketones has been widely described [12], only a limited number of reactions involve a Pd-catalyzed carbonylation step have been reported for the synthesis of vinyl ketones. Among them, the first synthetic application of the carbonylative Pd-catalyzed cross coupling reaction for the synthesis of α,β-unsaturated systems has been described in the seminal report by Suzuki and Miyaura in 1981 [70]. Alkenylboranes were reacted with carbon monoxide in the presence of palladium chloride and sodium acetate in methanol to give the corresponding α,β-unsaturated carboxylic esters with retention of configuration with respect to alkenylboranes in good yields (Scheme 14).

**Scheme 15:**
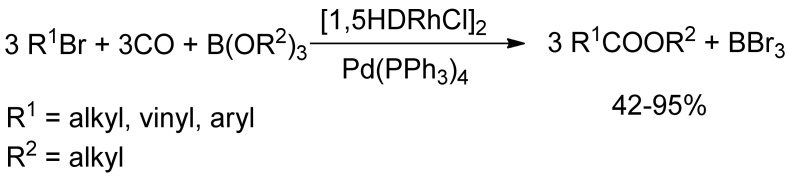
Alper’s Rh/Pd catalyzed carbomethoxylation of alkyl and vinyl bromides.

Few years later, Alper and coworkers [71] reported in a similar way the synthesis of α,β-unsaturated esters by reaction of alkyl, vinyl and aromatic bromides with organoborates and carbon monoxide, in the presence of catalytic bimetallic system of 1,5-hexadienerhodium(I) chloride dimer and tetrakis(triphenylphosphine) palladium (Scheme 15).

**Scheme 16:**
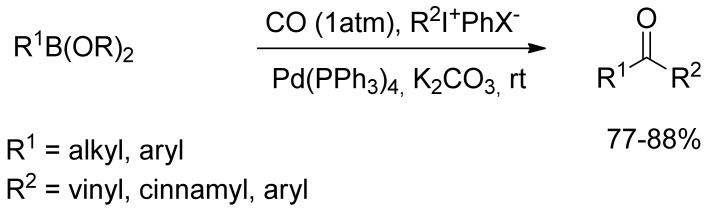
Hypervalent iodonium salts promoted synthesis of chalcone derivatives.

In 1998 Kang and coworkers [72] described the Pd-catalyzed carbonylative cross-coupling of organoboranes with hypervalent iodonium salts in the presence of Pd(PPh_3_)_4_ and potassium carbonate under an atmospheric pressure of carbon monoxide at room temperature (Scheme 16). The reaction was successfully applied with aryl-, alkenyl-, and alkynyliodonium salts at room temperature affording unsymmetric aromatic ketones and chalcones in moderate yields.

**Scheme 17:**
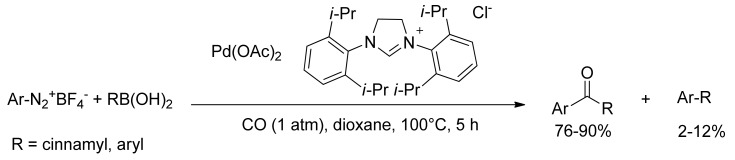
Andrus’s carbonylative coupling of aryl diazonium salts.

Andrus and coworkers reported the first example of carbonylative coupling of aryl diazonium tetrafluoroborate salts and aryl boronic acids to form aryl ketones and chalcones [73]. Palladium(II) acetate and *N*,*N*-bis-(2,6-diisopropylphenyl)dihydroimidazolium chloride (2 mol%) were used as catalytic system in the presence of an atmospheric pressure of carbon monoxide in 1,4-dioxane at 100 °C for 5 h (Scheme 17). Yields ranged from 76% to 90% with only minor amounts of biaryl coupling product observed (2%–12%).

**Scheme 18:**
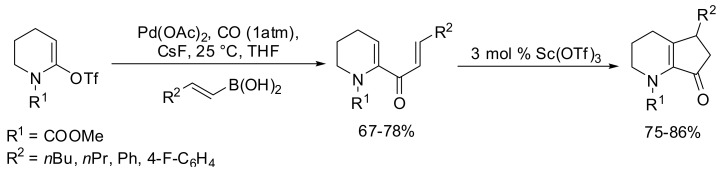
Pd-catalyzed carbovinylation of lactam-derived triflates.

The carbonylative Suzuki-Miyaura coupling of vinyl triflates with alkenylboronic acids has subsequently been reported for the first time by Occhiato and coworkers in 2006 [74]. A short series of alkenylboronic acids were tested as substrates for the SM carbonylative coupling with lactam derived triflate in the presence of 5% Pd(OAc)_2_ and 10% Ph_3_P as the catalyst, and CsF (3 equiv) in THF at 100 °C (Scheme 18). The best results were achieved by carrying out the reaction at room temperature, which avoided some degradation of the starting material. This efficient carbonylative Suzuki-Miyaura coupling reaction using alkenylboronic acids as the nucleophiles, provided 2-(*N*-methoxycarbonylamino)-1,4-pentadien-3-ones which have been employed as substrates for the Lewis acid-catalyzed Nazarov reaction.

This methodology for the preparation of unsymmetrical divinyl ketones was subsequently extended by the same research group to five- and six-membered heterocyclic systems namely lactams, lactones, and thiolactones [75,76,77]. Good to excellent yields (60%–86%) were obtained in particular with *N*-heterocyclic vinyl triflates, while good overall yields over two steps (50%–57%) were also obtained with lactone- and thiolactone-derived enol triflates.

## 4. SM with Masked Acyl Boronates

**Figure 2:**
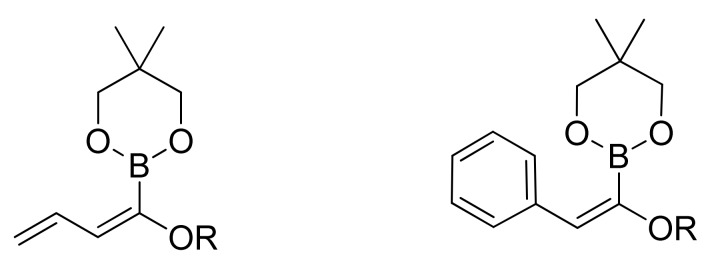
Alkoxydienyl and alkoxystyryl boronates.

The introduction of a masked acyl function onto a substrate that can be successively unmasked is a general and most widely applied method for acylation reaction. The use of masked acyl boronates offers a complementary alternative to carbonylative approaches. To this purpose alkoxy dienyl and alkoxy styryl boronates (Figure 2) have been synthesized and used as coupling partners in SM reactions. 

**Scheme 19:**
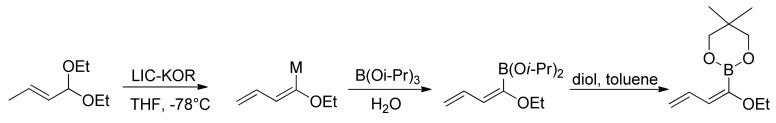
Synthesis of butadienyl boronic esters.

In our studies, we have developed a new synthesis of butadienylboronic esters, starting from α,β-unsaturated acetals, in the presence of LIC–KOR superbase (Scheme 19) [78].

**Scheme 20:**
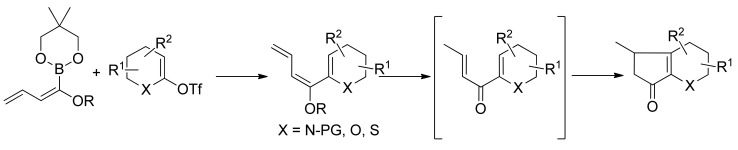
SM coupling between alkoxy dienyl boronates and heterocycle derived triflates.

The coupling products thus obtained can be used directly or after mild unmasking procedure as substrates for Nazarov electrocyclization (Scheme 20).

**Scheme 21:**
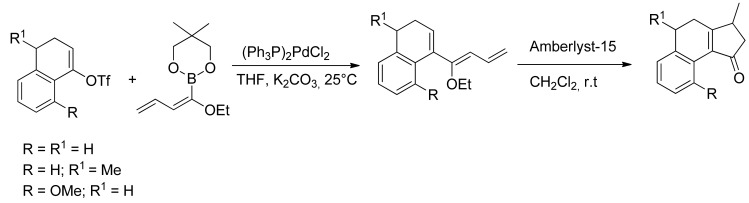
Synthesis of tricarbocyclic derivatives.

The ethoxydienylboronates thus formed can be used in an array of SM reactions with triflates or halides as coupling partners. In particular the reaction with α-tetralones-derived triflate, according to Suzuki-Miyaura conditions affords conjugated ethoxytrienes, which undergo cyclization in the presence of Amberlyst-15^®^, giving tricarbocyclic derivatives (Scheme 21) [78].

**Scheme 22:**
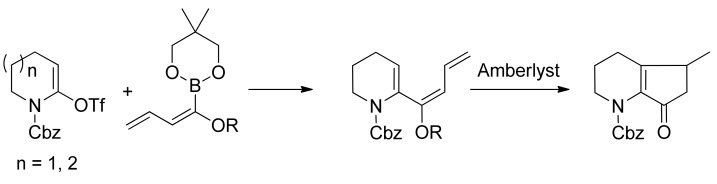
Synthesis of azepines.

Alkoxydienyl- and alkoxystyrylboronates were used for Pd-catalyzed cross-coupling reactions with lactam-derived vinyl triflates (Scheme 22). The hydrolysis of the coupling products with alkoxystyrylboronates provided the corresponding *N*-acyl-substituted 3,4-dihydro-(2*H*)-pyridines and 2,3,4,5-tetrahydroazepines in good to high yields. The hydrolysis of the coupling products with alkoxydienylboronates, performed in the presence of Amberlyst-15^®^, resulted in a Nazarov-type cyclization that afforded hexahydro[1]pyrindin-7-ones and 3,4,5,6,7,8-hexahydro-(2*H*)-cyclopenta[*b*]azepin-8-ones. This methodology represents a novel and efficient procedure for the preparation of these classes of azacyclic compounds [79].

**Figure 3:**
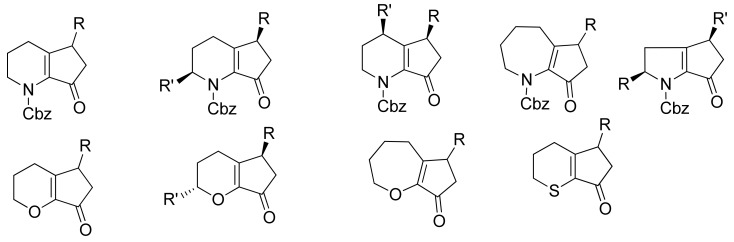
Representation of some of the scaffolds that can be synthesized through SM coupling between heterocyles derived triflates or phosphates and alkoxydienyl boronates.

The whole process has been extended to lactone and thiolactone-derived vinyl triflates and phosphates. As usual, if subjected to mild acidic hydrolysis, these compounds undergo a 4π*-*electrocyclization process which furnishes cyclopenta-fused *O*- and *S*-heterocycles in good yields. The scope of the work has been that of closely examining the role and effect of both the heteroatom and the heterocycle ring size on the outcome of the electrocyclization, as well as the torquoselectivity of this process. The presence of the heteroatom was essential in stabilizing the oxyallyl cation intermediate, thus allowing the reaction to occur [80]. The ring size was also a basic parameter in the cyclization step: five-membered azacycles required more drastic conditions to give 5–5 fused systems and did so only after an initial hydrolysis to the corresponding divinyl ketones. As for the torquoselectivity, with both 2-methyl and 4-methyl substituted lactam derivatives steric interactions seem to have a role in forcing the conrotatory process to take place in one sense only: allowing the synthesis of diastereomerically pure compounds to be realized [81,82]. Because different patterns of substitution on the heterocycle are compatible with the reaction conditions, the methodology developed could be very useful for the synthesis of natural products and biologically active compounds containing cyclopenta-fused *O*- and *N*-heterocycle moieties (Figure 3).

**Scheme 23:**
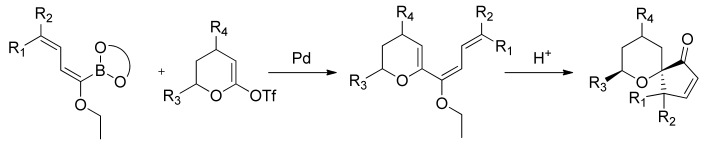
Synthesis of spyrocyclic ketones.

The methodology has been then applied to the synthesis of spirocyclic ketones. As a matter of fact, the Suzuki-Miyaura cross-coupling reaction between α-ethoxydienyl boronates and lactone-derived vinyl triflates affords functionalized 6-(1-ethoxy-1,3-butadienyl)dihydropyran derivatives that give functionalized spirocyclic ketones after hydrolysis under mild acidic conditions. The product distribution and the stereoselectivity of the process are strongly dependent on the substitution of both the ethoxydiene and dihydropyran moieties. High stereoselectivity is observed in the presence of a C2-substituent on the dihydropyran moiety (Scheme 23) [83], which has been explained in terms of transition state geometries.

**Scheme 24:**
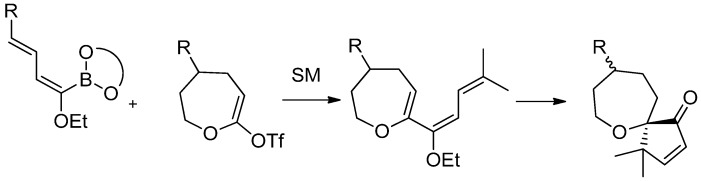
Synthesis of seven membered oxacycles.

The same synthetic approach was finally applied to seven-membered oxacycles, in order to obtain spirooxepanes. Coupling products were obtained by the usual Suzuki-Miyaura cross-coupling reaction and treated with Amberlyst-15^®^ affording the spiro products. As a consequence of the conformational flexibility of the seven-membered rings, a lack in diastereoselection has been observed in the cyclization process (Scheme 24).

**Scheme 25:**
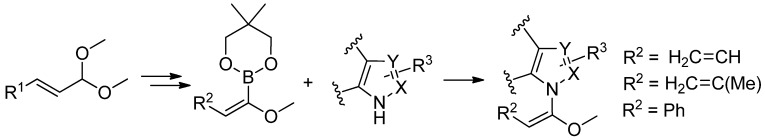
Coupling reactions between alkoxydienyl and alkoxystyrylboronates and azoles.

Furthermore, alkoxydienylboronates and alkoxystyrylboronates have been used in various copper mediated cross-coupling reactions with azoles. A variety of *N*-alkoxydienyl- and *N*-alkoxystyrylazoles have been synthesized under mild conditions. The process utilizes Cu(OAc)_2_ in CH_2_Cl_2_ at room temperature in the presence of CsF (Scheme 25) [84].

## 5. Acylation of Heterocycles by SM in the Synthesis of Natural Products

Nowadays, there are numerous applications of palladium catalysts in the preparation of pharmaceuticals, agrochemicals, and also advanced materials both on laboratory and industrial scale [85]. Among the wide number of Pd-catalyzed coupling reactions, the direct acylation of heterocycles and the carbonylation reactions also have experienced impressive improvements for the construction of diaryl ketones (see above) or substituted heteroaromatic moieties which constitute important building blocks of polymers [86], ligands [87], natural products as well as some biologically active pharmaceuticals [88]. In this last section we provide herein an overview of the direct acylation of heterocycles applied as key synthetic step in the preparation of natural products.

### 5.1. SM Acylation Reaction for the Synthesis of Strigolactones (SLs)

**Figure 4:**
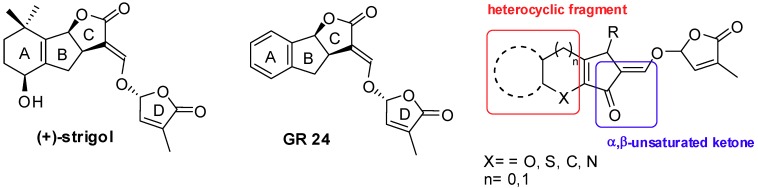
Naturally occurring strigol with reference compound GR 24 (left) and our target analogue (right).

Since 2008 our research has been focused on the total synthesis of a family of plant hormones called strigolactones (SLs). This class of molecules proved to play multiple roles in regulating the rhizosphere: they are involved in the signaling between plants and symbiotic fungi and in the signaling of other different biological systems such as the germination of parasitic plants seeds, fungi hyphal branching, bacterial mitosis and plant shoot branching [89,90]. The SLs chemical structure is generally based on an ABC tricycle core linked to a D cycle by an enol ether bridge. Our work in this field has been mainly focused on the development of a innovative synthetic procedure aimed at the extension of the conjugated system to the ABC framework across an heterocyclic B ring and the replacement of the carboxylic moiety (present on the C ring) with a more reactive CO moiety (Figure 4) [91,92].

**Scheme 26:**
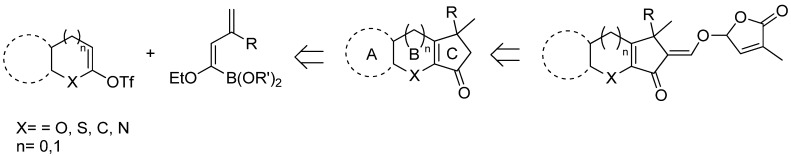
Retrosynthetic approach to SL’s ABC core through SM-coupling of dienyl boronate derivatives.

To this purpose, a retrosynthetic approach to the tricyclic framework ABC where a SM-coupling of a masked acyl anion with a heterocyclic triflate has been designed and exploited as the key step for the construction of the desired scaffold, as shown in Scheme 26.

**Scheme 27:**
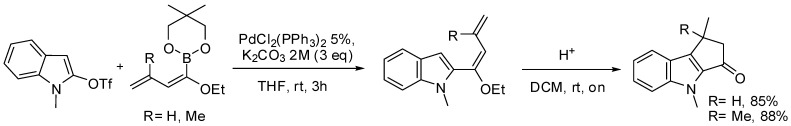
SM coupling of dienyl boronates for the synthesis of strigolactones ABC core.

Thus, a simple Suzuki coupling between indolyl triflate and alkoxy dienyl boronates in the presence of palladium(II) catalyst and aqueous potassium carbonate in THF afforded indolyl functionalized dienes in good yields at room temperature (Scheme 27) [89,90]. Upon treatment under mild acidic conditions, the alkoxy vinyl ethers unmasked the corresponding vinyl ketones that underwent Nazarov cyclization leading to the desired tricyclic core.

**Scheme 28:**
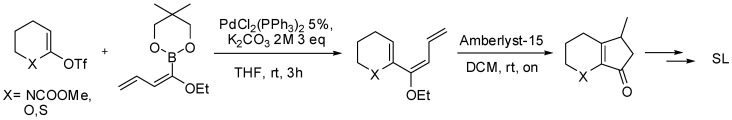
Pd-catalyzed alkenylation of lactones and lactames applied to the synthesis of bicyclic SL’s analogues.

The same procedure was then extended to the synthesis of new SL analogues missing the A ring [89]. To this purpose *N*-protected δ-valerolactam, δ-valerolactone or thiolactone were successfully coupled with the dienyl boronate under the same reaction conditions. The coupling products were then subjected to a Nazarov cyclization with Amberlyst-15 to afford the bicyclic systems (Scheme 28) that were subsequently converted to the corresponding strigolactone derivatives.

**Scheme 29:**
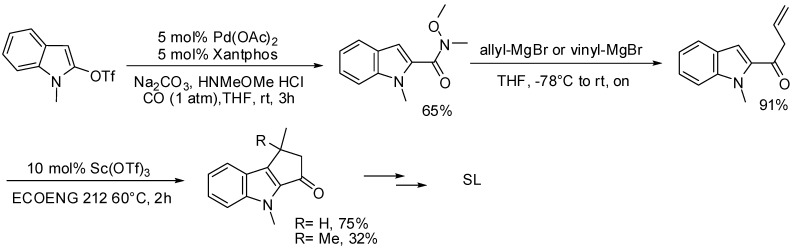
Acylation of indolyl triflates applied to the synthesis of SL’s tricyclic core.

As an alternative procedure to the synthesis of the SL’s bi- or tricyclic core, we have also exploited a classic carbonylative approach for the preparation of heterocyclic Weinreb amides by means of a palladium catalyzed coupling of indolyl triflate with *N*-methoxy-*N*-methylamine hydrochloride [89]. The resulting amide was successfully coupled with allylmagnesium bromide and the resulting dienone, which possesses the suitable electronic arrangement to undergo an acid catalyzed Nazarov reaction, was subsequently subjected to ring closure under mild acidic conditions (Scheme 29).

### 5.2. SM Coupling Applied to the Synthesis of Other Natural Products

**Scheme 30:**
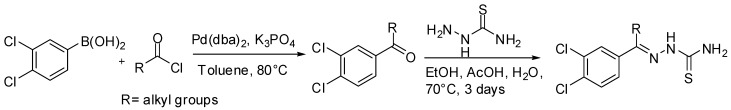
Guy’s synthesis of thiosemicarbazone inhibitors.

Despite the direct acylation of heterocycles has not been widely described, few examples of natural products syntheses involving SM-couplings acylation steps have been reported in the literature so far. The first example, reported by Guy and coworkers [93,94], is based on the discovery of potent thiosemicarbazone inhibitors of rhodesain [95] and cruzain [96] for the treatment of Chagas’ disease and sleeping sickness. Thiosemicarbazones have potent activity against rhodesain [97], protease of the papain-like family expressed by the protozoan parasite *Trypanosoma brucei* which causes Human African trypanosomiasis (HAT), a major health concern in sub-Saharan Africa with an estimated 50,000 cases and 60 million at risk of infection. In the synthetic sequence to rhodesain and cruzain inhibitors described by Guy, the key ketone intermediate was prepared by a SM coupling between an appropriate acyl chloride and a boronic acid by means of Pd(dba)_2_ in toluene. Finally, an acid catalyzed reaction with thiosemicarbazide afforded the target thiosemicarbazones in moderate to good yields (Scheme 30).

**Figure 5:**
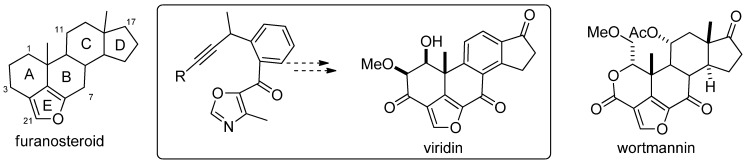
Furanosteroid class of PI3-kinase inhibitors.

The second example we are presenting herein is focused on the synthesis of furanosteroids, performed by Jacobi and Sessions in 2006 [98]. The furanosteroids are a class of novel pentacyclic fungal metabolites that share in common a furan ring, bridging positions 4 and 6 of the steroid skeleton (Figure 4) [98]. Members of this class have attracted attention for many years because of their potent antiinflammatory and antibiotic properties [99] and more recently because of their ability to selectively block certain intracellular signaling pathways associated with cell growth and development [100,101]. Prominent members include those of the viridin and wortmannin families (Figure 5). In his report, Jacobi described the synthesis of viridin via an acyl oxazole intermediate, which its synthetic key step required a SM acylation.

**Scheme 31:**
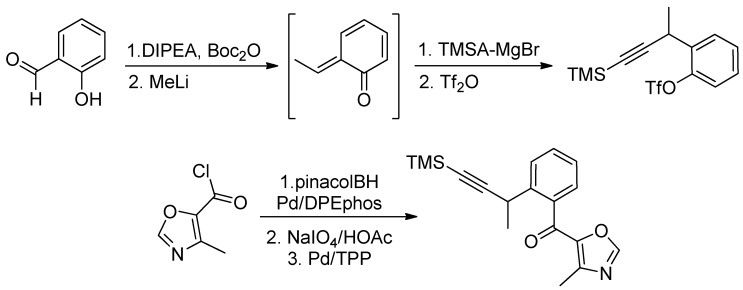
Jacobi’s synthesis of furanosteroid viridin’s core.

The salicylaldehyde was converted to the Boc derivative and next, the treatment with MeLi generated the reactive *o*-quinone methide, by a pathway involving nucleophilic addition to the aldehyde, followed by intramolecular transfer of the Boc group and conjugated elimination. Quenching with the Grignard reagent derived from trimethylsilylacetylene (TMSA) followed by triflation gave the corresponding alkyne in 74% yield. Finally, the TMS-alkynyl acyl oxazole was obtained in a three-step sequence starting from triflate via an elaboration of the corresponding boronic acid and a Suzuki coupling with the readily available acyl chloride (Scheme 31).

In the world of natural products diaryl ketones are important structural motifs which are present in a large number of biological and medicinal interest compounds (e.g., cotoin, papaveraldine, suprofen, ketoprofen), and the synthesis by direct acylation in SM-couplig reactions has already been described in the previous sections of this survey.

**Scheme 32:**
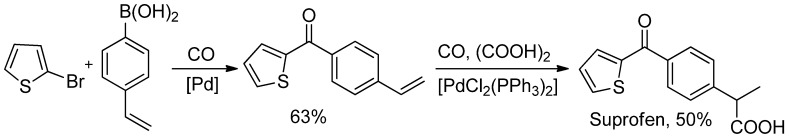
Beller’s two-steps synthesis of Suprofen.

Differently from direct acylation of heterocycles, more examples about carbonylative SM reaction applied to the synthesis of natural products are available. For example, in 2008 Beller and co-workers [102] synthesized suprofen, a commercial nonsteroidal anti-inflammatory drug, in only two-steps (Scheme 32).

Thus, 2-bromothiophene was reacted with 4-vinylphenylboronic acid in the presence of CO to give exclusively the desired diarylketone in 63% yields. Hence, 4-vinylphenyl 2-thienyl ketone was easily hydroxycarbonylated [103] in one step to suprofen.

**Scheme 33:**
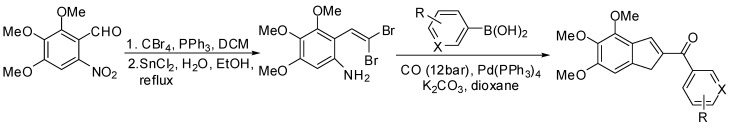
Synthesis of Combrestatin A4 analogues.

Recently, Florent *et al.* [104] synthesized novel heterocyclic combretastatin A4 analogues. Combretastatin A4 (CA4) is a natural product isolated by Pettit *et al.* from the South African willow tree *Combretum caffrum* [105]. It strongly inhibits tubulin polymerization and the proliferation of murine and human cancer cell lines (Scheme 33) [106].

The required trimethoxyanilines were easily prepared in a two-step sequence (Ramirez–Corey–Fuchs olefination [107] followed by reduction of the nitro group) from the corresponding *o*-nitrobenzaldehyde derivatives [108]. The domino reaction was then performed heating the dibromoolefine and phenylboronic acid at 85 °C for 24 h, and the desired 2-aroylindole was isolated in satisfactory yield (65%), opening the possibility to get a novel series of CA4 analogues.

**Scheme 34:**
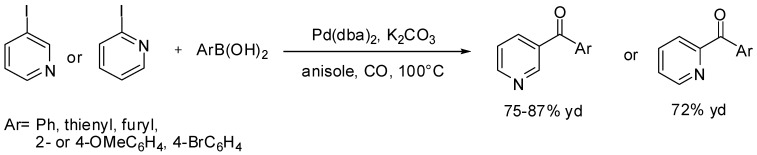
Yang and Xue’s synthesis of heteroaryl-substituted pyridine derivatives.

Finally, Yang and Xue [109] and Bhanage [110] synthesized heteroaryl-substituted pyridine derivatives by means of Suzuki carbonylative reactions (Scheme 34). These molecules are a very important family of compounds with several applications in diverse areas of chemistry such as metal-coordination complexes [111,112], pharmaceutical agents [113,114], and molecular electronic device materials [115,116,117].

## 6. Conclusions

The Suzuki palladium-catalyzed cross-coupling reaction of organoboron reagents with organic halides or pseudohalides to form biaryl derivatives has emerged over the past two decades as an extremely powerful tool in organic synthesis. More recently, the SM coupling has been exploited as an efficient method to introduce acyl functionalities on a target substrate. The intent of this short review is to give an overview of the most recent approaches found in the literature to reach this goal. Three different strategies can be pinpointed. The first of all relies on the coupling between organoboron reagents and acyl derivatives (acyl chloride, anhydrides, chloroformates and carbamoyl chlorides). The palladium-catalyzed three-component cross-coupling of aryl halides, carbon monoxide, and boronic acids, which is closely related to the Suzuki reaction, also provides convenient access to asymmetric ketones. However, the main drawback of the carbonylative Suzuki reaction often lies in the formation of significant amounts of the direct coupling product without carbon monoxide insertion, particularly with electron-deficient aryl halides. Finally, boronic derivatives with masked acyl functions (alkoxy dienyl or styryl boronates) can be used as a tool to introduce a masked acyl function by means of a traditional SM coupling, followed by a mild unmasking step. A selection of natural product syntheses in which SM acylation represents a key step is presented in the last section.
